# Malnutrition and Infection: Complex Mechanisms and Global Impacts

**DOI:** 10.1371/journal.pmed.0040115

**Published:** 2007-05-01

**Authors:** Ulrich E Schaible, Stefan H. E Kaufmann

## Abstract

The authors discuss current concepts and controversies surrounding the complex influences of malnutrition on infection and immunity, and point to practical consequences of countermeasures in acute malnutrition.

*“I am not interested in the bloody system! Why has he no food? Why is he starving to death?”*

*Bob Geldof in* The Observer, *1985*

Activation and sustenance of immune responses during infection requires increased energy consumption. Protein energy malnutrition (PEM) is a critical, yet underestimated factor in susceptibility to infection, including the “big three” infectious diseases: HIV/AIDS, tuberculosis, and malaria. In this article, we discuss current concepts and controversies surrounding the complex influences of malnutrition on infection and immunity, and point to practical consequences of countermeasures in acute malnutrition. We call for new strategies to overcome worldwide morbidity and mortality caused by chronic malnutrition in impoverished countries and by the newly emerging public health threat of overnutrition in industrialized societies.

## Background

In response to infection, the immune system first executes innate and then subsequently acquired host defense functions of high diversity. Both processes involve activation and propagation of immune cells and synthesis of an array of molecules requiring DNA replication, RNA expression, and protein synthesis and secretion, and therefore consume additional anabolic energy. Mediators of inflammation further increase the catabolic response. Studies in a simple system, involving measurement of the survival of malnourished bumblebee workers, showed that the energy cost of immunity further impairs fitness [1]. Consequently, the nutritive status of the host critically determines the outcome of infection.

**Figure 1:**
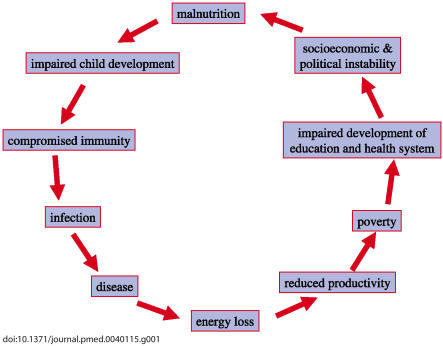
Protein Energy Malnutrition Increases Prevalence of Infection, Leading to Energy Loss for the Individual On the community level, this burden reduces productivity, including food production, and perpetuates the relentless spiral of further malnutrition, infection, disease, poverty, and socioeconomic and political instability.

Apart from deficiencies in single nutrients, such as vitamins, fatty acids, amino acids, iron, and trace elements, undernourishment based on PEM greatly increases susceptibility to major human infectious diseases in low-income countries, particularly in children [2–4]. Malnutrition is responsible, directly or indirectly, for 54% of the 10.8 million deaths per year in children under five and contributes to every second death (53%) associated with infectious diseases among children under five years of age in developing countries [5]. Infection causes energy loss on the part of the individual, which reduces productivity on the community level and perpetuates the alarming spiral of malnutrition, infection, disease, and poverty (Figure 1).

## Malnutrition and Infection

### Malnutrition increases risk of infection

PEM is a common cause of secondary immune deficiency and susceptibility to infection in humans (Table 1). This causal relationship is further supported by animal studies.

**Table 1:**
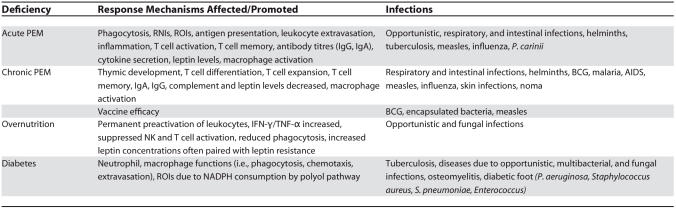
Conditions of Under- and Overnutrition and Their Influence on Host Defense Functions

Severe PEM in children is clinically defined as less than 70% weight-to-height and/or the appearance of pitting edema on both feet, described as either marasmus, a chronic wasting condition, or kwashiorkor, characterized by edema and anemia. Children with kwashiorkor often suffer from marked skin infections. Severe malnutrition during childhood affects thymic development, which compromises immunity in children by a long-term reduction of peripheral lymphocyte counts [6]. This immunodeficiency represents a key factor in susceptibility to infections and has therefore been termed nutritionally acquired immunodeficiency syndrome [7]. In severely malnourished patients, both acquired immunity—i.e., lymphocyte functions—as well as innate host defense mechanisms—i.e., macrophages and granulocytes—are affected. Diminished immune functions render undernourished patients more susceptible to infections, notably those by opportunistic pathogens commonly prevalent in patients with HIV/AIDS [2–4,8,9]. The opportunistic fungus Pneumocystis carinii, frequently diagnosed in patients with AIDS, was repeatedly identified in malnourished children after the Second World War [9]. Noma is an opportunistic infection in children between one and four years with PEM, which occurs worldwide, but is most common in sub-Saharan Africa. The infection evolves from gingival inflammation to orofacial gangrene and is commonly preceded by other infections such as measles, malaria, severe diarrhea, and necrotising ulcerative gingivitis. Noma coincides with the period of linear growth retardation in malnourished children [10].

In addition to promoting acute and chronic infections, PEM impairs the linear growth of children, leading to a further reduction in food intake, nutrient absorption, direct or catabolic nutrient losses, and increased metabolic requirements. It has been suggested that acute phase response and proinflammatory cytokines directly affect the bone remodelling required for longitudinal growth [11].

Correlation of malnutrition and growth retardation allows assessment of the individual nutritional state, which is usually measured as mid-upper arm circumference or body mass index (BMI). BMIs are given either as weight-for-height to indicate acute PEM (wasting), or as weight-for-age (underweight) or height-for-age (stunting), correlations for chronic PEM. A study in Kenya found a significant association between HIV infection and lower mid-upper arm circumferences and serum albumin concentration, another measure of malnutrition, but found no such association with BMI [12]. Independent of HIV, socioeconomic factors and severity of tuberculosis are important correlates of acute PEM or wasting [12].

**Infection itself contributes to malnutrition.** The relationship of malnutrition on immune suppression and infection is complicated by the profound effects of a number of infections on nutrition itself. Examples of how infections can contribute to malnutrition are: (1) gastrointestinal infection can lead to diarrhea; (2) HIV/AIDS, tuberculosis, and other chronic infections can cause cachexia and anemia; and (3) intestinal parasites can cause anemia and nutrient deprivation [13].

Stimulation of an immune response by infection increases the demand for metabolically derived anabolic energy and associated substrates, leading to a synergistic vicious cycle of adverse nutritional status and increased susceptibility to infection. Under inflammatory conditions such as sepsis, mediators increase the catabolic disease state characterised by enhanced arginine use. Furthermore, arginase is induced during infection and uses up arginine as substrate. It has been suggested that depletion of this amino acid impairs T cell responses [14], and exceeding the body’s arginine production leads to a negative nitrogen balance [15].

A study in Nigeria found that the severe metabolic demands made during acute measles infection further deteriorated the condition of malnourished children, leading to further weight loss, wasting, and reduced serum levels of essential amino acids [16]. Increased energy consumption due to immune responses may also affect the efficacy of live attenuated vaccines in populations ridden with PEM.

Arginine treatment has been shown to improve nitrogen balance and lymphocyte function and stimulate arginine transport in the liver. These benefits have made arginine an essential constituent of immunonutritive formulas currently in use for critically ill patients.

PEM is an important health determinant for critically ill patients and increases susceptibility to infections in malnourished elderly patients and patients with anorexia. A large and strictly controlled inpatient study in France pinpoints malnutrition as an independent risk factor for nosocomial infections, which account for 6%–10% of all in-hospital deaths worldwide [17]. Accordingly, nutritive management has to become an elementary part of intensive health care. In summary, nutritional quality and composition are pivotal for anti-infectious immunity.

## Malnutrition Affects Immunity

Severe protein malnutrition in newborns and small children causes atrophy of the thymus with reduced cell numbers and subsequently ill-developed peripheral lymphoid organs, i.e., lymph nodes and spleen [6]. This causal chain leads to long-lasting immune defects characterized by leucopenia, decreased CD4 to CD8 ratio and increased numbers of CD4/CD8 double-negative T cells, and, therefore, the appearance of immature T cells in the periphery. Malnourished children suffer in greater proportion from respiratory infections, infectious diarrhea, measles, and malaria, characterized by a protracted course and exacerbated disease. These malnourished children present with diminished functional T cell counts, increased undifferentiated lymphocyte numbers, and depressed serum complement activity (Table 1).

Reduced antibody responses to polysaccharide antigens of encapsulated bacteria such as Streptococcus pneumoniae and Haemophilus influenzae exacerbate susceptibility to these pathogens [2,4,18]. Moreover, immune defense at the epithelial barrier of the undernourished host is compromised due to altered architecture of the gut mucosa, such as flattened hypotrophic microvilli, reduced lymphocyte counts in Peyer’s patches, and reduced immunoglobulin A (IgA) secretion [7]. Availability of complement components is restricted by malnutrition, thereby affecting the capacity of professional phagocytes to engulf and eliminate pathogens. In mice with experimental PEM, phagocytosis and production of reactive oxygen intermediates (ROIs) and reactive nitrogen intermediates (RNIs) by macrophages is diminished, as is antigen presentation to T cells by dendritic cells [19]. Temporary PEM in mice challenged by experimental peritonitis resulted in impaired immune cell migration and extravasation, as indicated by reduced numbers of CD11b/CD18-positive cells at the site of infection, probably involving lower concentrations of the chemokine MIP-2.

Peripheral T lymphocytes from infected children with PEM had lower expression of the activation marker CD69, and predominantly showed an intermediate (CD45RA^low^/CD45RO^low^) rather than a memory phenotype (CD45RO^high^) when compared to healthy donors [20,21]. These T cells were biased towards type 2 T helper cell (Th2) responses, represented by decreased IFN-γ/IL-2 (type 1 T helper cell [Th1]) and increased IL-4/IL-10 (Th2) production [22]. Experimentally undernourished weanling mice had predominantly T cells of the naïve quiescent phenotype (CD45RA^+^/CD62L^+^) [23,24]. In these mice, IFN-γ- responses were depressed and IL-10 and the Th2-associated antibody, IgE, were increased, while IL-4 production remained normal [25].

These findings, however, should not be taken to suggest that PEM generally biases towards Th2 responses. Rather, PEM appears to alter immune responses, thus hampering protective immunity of any type. Protective T cell responses against helminth infections are predominantly of the Th2 type comprising IL-4 production, expansion of eosinophils, and IgE secretion. However, malnourished children are deficient for protective IgE antibodies against Ascaris lumbricoides [26,27]. By suppressing such responses in mice, PEM increases susceptibility to infection with the intestinal parasite, Heligmosomoides polygyrus [28]. Malnourished children suffering from helminth infections have high concentrations of total IgE. Yet these antibodies are neither worm-specific nor protective, and their memory T cells do not recognize helminth antigens [27,28].

## Malnutrition and Tuberculosis: Yesterday and Today

Malnutrition is generally appreciated as a major risk factor in the onset of active tuberculosis [9]. This notion is largely based on historical reports but also on more recent experimental animal studies.

One of the major disease burdens globally, tuberculosis is a well-documented example of the way in which malnutrition leads to worse disease outcomes. During the First World War, Denmark was affected by a tuberculosis epidemic similar to that prevailing in countries at war. The Danish tuberculosis epidemic could be explained by widespread malnourishment, since the export of meat, fish, poultry, and dairy products meant that food was scarce inside the country. This tuberculosis epidemic plummeted once the German blockade of Denmark was established and food became available to the Danish population again, but the epidemic continued in other countries [9].

**Five Key Papers on the Link Between Malnutrition, Immunity, and Infection**

**Ing et al., 2000 [28]** Employing an animal model for helminth infection, this study shows that malnutrition interferes with protective immunity.

**Cegielski et al., 2004 [9]** This paper summarizes, for the first time, clear evidence of the link between malnutrition and tuberculosis.

**Zarkesh-Esfahani et al., 2004 [60]** The authors show the link between leptin-induced TNF- and neutrophil activation.

**Turnbaugh et al., 2006 [66]** This paper puts a new component on to the map of research on overnutrition: the gut flora’s influence on energy uptake.

**Tectonidis, 2006 [76]** This paper points out a quick and practical approach to remediate acute malnutrition.

A comparative radiographic survey of prisoners of war held in German camps during the Second World War under similar living conditions found a tuberculosis prevalence of 1.2% versus up to 19.0% among the British and Russians, respectively, with more severe outcomes in the latter. This difference in prevalence and severity is probably a direct consequence of the fact that only the British prisoners received—in addition to the regular prison diet—a Red Cross supplement of 30 grams of protein and 1,000 kilocalories per day. This causal relationship is in line with the positive correlation of below average BMI with increased risk of pulmonary tuberculosis [9].

**Unexplored Research Topics on the Link Between Malnutrition and Infection**

1. Control of energy uptake by the host through microbial communities in the gut.
2. Changes of gut flora in malnutrition and influence of probiotics.
3. Regulation of energy requirement and food uptake during immune responses.
4. Role of cytokines in regulating nutrient uptake.
5. Crosstalk between immune (cytokines) and nutrition-related “hormones” (e.g., leptin).
6. Tissue-specific loss of nutrients during infection, inflammation, and immunity.
7. Efficacy of vaccines and drugs given under acute and chronic PEM conditions.
8. Identification of essential immunonutrients and development of food supplements easily accessible for societies with chronic PEM.
9. Schedules for sustainable local food production and safe water supplies by societies suffering from PEM.
10. Efficacy of additives to drinking water and food to prevent infection.
11. Engineering nutritionally enhanced food to prevent infections (e.g., rice, sorghum)
12. Bringing it to the people: Improved infrastructures for food distribution in developing countries.

More contemporary reports provide further support that malnutrition has an impact on the clinical outcome of tuberculosis [29]. A statistically significant number of patients with tuberculosis were malnourished in a recent study in Sri Lanka and skin test reactions for tuberculosis were negatively affected by malnutrition [30,31]. Hence, in poor settings, nutritional measures should be considered as an adjunct to anti-tuberculosis drug treatment.

Animal experiments, mainly in the guinea pig tuberculosis model, document detrimental consequences of chronic PEM on immunity to Mycobacterium tuberculosis. In these experiments, lymphocyte stimulation as well as secretion of the Th1 cytokines IL-2, IFN-γ, and TNF-α, involved in control of M. tuberculosis, were significantly reduced in animals with PEM [9]. Moreover, macrophages from such animals produced more transforming growth factor β (TGFβ), which further suppresses T cells and inflammation [32,33]. A study in murine tuberculosis reached similar conclusions and additionally found that malnourished mice showed hampered production of RNIs, which act as critical effectors against tuberculosis in mice. Consequently, malnourished mice suffered from higher bacterial burdens and died earlier of infection [34]. Finally, efficacy of BCG vaccination against tuberculosis was profoundly reduced in malnourished guinea pigs as compared to normally fed animals, due to impaired T cell priming and function [9,35].

## Malnutrition, Leptin, and Immunity

Leptin is a central mediator connecting nutrition and immunity. Levels of the pleiotropic hormone leptin, which regulates satiety, are reduced in patients with PEM. Leptin concentrations correlate with body fat mass and are quickly reduced by fasting [36]. Leptin is a 16 kDa α-helix type protein similar to the cytokines IL-6 and IL-12, and is mainly secreted by adipose tissue. At least six receptors representing different splice forms encoded by one gene are broadly distributed on different cell types. The isoform is not only full-length ObR_b_ expressed in the hypothalamus, but is also prevalent on lymphocytes and macrophages [36,37]. Leptin binding activates immune cells via the to ObR_b_ JAK-2/STAT-3 and the MAPK pathway and induces TNF-α, IL-6, and IL-12 secretion in macrophages. Leptin stimulates naïve T cells (CD45RA^+^) but blocks proliferation of memory T cells (CD45RO^+^). Concomitantly, leptin promotes IFN-γ secretion by memory T cells, inhibits Th2 responses [38,39], and induces activation markers (CD69, CD25, and CD71) [40]. Apart from inducing lymphopoiesis, leptin seems to deliver survival signals to T cells by upregulating anti-apoptotic proteins T-bet and Bcl-x_L_ [36].

Active tuberculosis is associated with cachexia, weight loss, and low serum concentrations of leptin [41–44]. Moreover, leptin-deficient mice are more susceptible to M. tuberculosis than wild-type mice, and T cell numbers, including those producing IFN-γ, are reduced in infected lungs, suggesting that leptin contributes to protection against tuberculosis [45]. However, a causative correlation between severity of tuberculosis and leptin is not fully established, and leptin concentrations do not predict wasting in human tuberculosis [44].

Malnutrition causes immunosuppression through a variety of mechanisms, including the involvement of leptin and the hypothalamic-pituitary-adrenal axis. PEM reduces leptin concentrations and increases serum levels of stress hormones, i.e., glucocorticoids [2,4,46–48]. Thus, it is likely that the hypothalamic-pituitary-adrenal axis plays a critical role in malnutrition-associated immune deficiency.

In well-nourished people, infection and inflammation increase leptin levels in an IL-1-dependent manner and increase glucocorticoid concentrations, which subsequently can control inflammation [40,49,50]. Under conditions of PEM and low leptin concentrations, glucocorticoids impair macrophage functions by decreasing NF-kB translocation into the nucleus [49]. Macrophages from mice with experimental PEM are less sensitive to activation with lipopolysaccharides, partly due to decreased NF-kB translocation. Their ability to engulf pathogens and to produce cytokines and ROIs is impaired [51–54]. However, the suggestion that malnutrition suppresses macrophage functions due to elevated glucocorticoid levels was not supported by a recent study [55]. Further experiments are required to identify the mechanisms connecting PEM and immunosuppression.

## Overnutrition and Immunity

The hormonal connection between immunity and nutrition becomes equally evident in nutritional dysregulatory eating disorders such as obesity, which is becoming alarmingly common in high-income countries, notably in the United States and United Kingdom, and is also spreading to transitional societies at an unexpectedly high speed.

Obesity in humans is correlated with high concentrations of leptin, often associated with leptin resistance [36]. Patients with obesity present with increased TNF-α production, altered T cell subset ratios, repressed T cell responses, and higher incidence of infectious diseases, all of which can be reversed by weight loss (Table 1) [56–58]. Diet-induced or inherited obesity in rodents causes NK and T cell suppression and increased TNF-α secretion [56,57,59]. Leptin-induced production of proinflammatory cytokines by macrophages causes neutrophil activation and TH1-derived IFN-γ secretion [60–62]. The obese phenotype in leptin-deficient *ob/ob* mice is also associated with diminished circulating T cells, reduced T cell responses, and lymphoid atrophy [40]. Although seemingly in a committed stage, macrophages from *ob/ob* mice have reduced phagocytic activity [40]. Furthermore, the natural ligand of the secretagogue receptor of the pituitary gland, ghrelin, which regulates fat storage and consumption, is directly linked to immune functions by its counteraction of leptin-induced activation of monocytes and T cells [63,64].

Recently, the cytokine IL-18, which usually drives TH1 responses in synergy with IL-12, has been linked to obesity. IL-18 knockout mice became obese through overeating and resistant to insulin through increased gluconeogenesis in the liver [65]. Consequently, intracerebrally administered IL-18 inhibited food intake. Another study demonstrated that a differential gut flora with distinct metabolic requirements was found in obese versus lean humans as well as mice. Even more intriguingly, when transferred to germ-free mice, the “obese” but not the “lean” gut flora caused an increase of total body fat [66]. These reports add two more components to the already complex relationship of nutrition, inflammation, immunity, and infection: cytokine patterns and gut flora compositions.

Thus, regulation of food uptake and storage is closely intermingled with immune functions. However, a higher nutrient uptake may also be beneficial for the host response, likely due to higher energy requirements, as illustrated by a recent study showing that a cholesterol-rich diet accelerates clearance of bacilli during the treatment of tuberculosis [67,68].

Diabetes mellitus is a hormonally regulated metabolic disease which affects immunity to infection (Table 1). Neutrophils and macrophages from patients with diabetes have suppressed functions, including phagocytosis, generation of ROIs, chemotaxis, and extravasation. T cell activation is also affected, as evidenced by reduced delayed-type hypersensitivity reactions [69]. Generation of ROIs requires NADPH, which is consumed by the polyol pathway for glucose metabolism. Patients with diabetes are more prone to diseases caused by Staphylococcus aureus and M. tuberculosis, and show higher mortality and morbidity from infections with S. pneumoniae and influenza virus [69]. In patients with diabetes, diseases due to urogenital tract and opportunistic infections by Enterococcus, Mucor mucedo, and Candida albicans are common. Pseudomonas aeruginosa is a frequent cause of abscesses in patients with diabetes, as are polybacterial infections causing skin ulcers (diabetic foot), and severe osteomyelitis [69,70]. There is clear evidence that proper control of hyperglycemia improves immune functions and resistance to infection.

## Outlook

Extrapolating the studies discussed, malnutrition can be considered a major risk factor for morbidity and mortality worldwide due to infections with bacterial, viral, and protozoal agents [2,8,9]. This causal relationship was suggested in the US Surgeon General’s Report in 1988 [71]. With more than 842 million chronically malnourished people worldwide [72], we agree with the notion that “…malnutrition may account for a greater population-attributable risk of tuberculosis than HIV infection, and certainly a much more correctable one” [9].

In the context of what is known as the 10/90 gap (10% of global health research funding is being targeted to health problems that account for 90% of the global disease burden) [73,74], research on infection and malnutrition are highly warranted for scientific, economic, and ethical reasons [75].

To conquer malnutrition, cost-efficient and practical approaches need to be established. Measures to counteract acute malnutrition are now available and were successfully applied in 2005 when Niger was affected by a famine. The crisis did not come as a surprise to the consortium of stakeholders, i.e., the government of Niger, its international partners, and the Famine Early Warning Systems Network of the US Agency for International Development. To avoid disturbance of the market and long-term development goals, food was sold to starving people for too high a price instead of being freely distributed. The catastrophe became apparent as vast numbers of malnourished children were brought to medical stations. Ready-to-use therapeutic food was delivered by doctors from Médecins Sans Frontières as an outpatient measure (community therapeutic care) with enormous success [76].

Usually children are hospitalized under such circumstances and given milk products as therapeutic food. Outpatient treatment during emergencies, however, decreases (1) duration of maternal absence from the family, thereby limiting children’s risk of malnourishment, (2) time needed to establish treatment centers, and (3) risk of spreading nosocomial infections among hospitalized children in a limited number of overcrowded places. New therapeutic food formulations with balanced contents of macro- and micronutrients, which are ready to use and do not need a clean water supply for their preparation, are important prerequisites for such rapid aid measures. In the past, life-threatening respiratory infections, diarrhea, and malaria were frequent complications requiring short-term inpatient anti-infectious treatment. Under the emergency conditions of the Niger famine in 2005, the measures employed by Médecins Sans Frontières kept child mortality at the rate of non-famine periods. Thus, there is a precedent of effective interventions for acute malnutrition in an emergency to avoid subsequent infections.

This measure, however, is unlikely to minimize mortality and morbidity due to *chronic* malnutrition worldwide. Further research and development in diverse areas ranging from biomedicine to public health are required to stop the downward spiral of chronic malnutrition, infection, disease, and reduced economic productivity in impoverished societies with the consequences of migration and economical and political instability or war (Figure 1). Diseases resulting from overnutrition in industrial societies are of equal concern and similar conditions are already spreading to developing countries. Under- and overnutrition and diet-related chronic diseases represent a critical risk factor for more than half of the world’s diseases and incur hundreds of millions of dollars in public expenditure [5], requiring the immediate attention of biomedical science and public health agencies alike.

## Supporting Information

Alternative Language Abstract S1 Translation of article summary into Russian (21 KB DOC).

Alternative Language Abstract S2 Translation of article summary into Spanish (20 KB DOC).

Alternative Language Abstract S3 Translation of article summary into French (20 KB DOC).

## Abbreviations

BMI: body mass index
lg: immunoglobulin
PEM: protein energy malnutrition
RNI: reactive nitrogen intermediate
ROI: reactive oxygen intermediate
TH1: type 1 T helper cell
TH2: type 2 T helper cell
