# Supplementary material for: Malnutrition and Infection: Complex Mechanisms and Global Impacts
Source: PLoS Med. 2007 May 1;4(5):e115. doi: 10.1371/journal.pmed.0040115 (PMC1858706; doi:10.1371/journal.pmed.0040115)
Supplement: Supplementary file 1 [file pmed.0040115.sd001.doc]

Активация и поддержание иммунных реакций во время инфекции требует повышенного потребления энергии. Недостаток в пищевом белке является определяющим и в то же время недооцениваемым фактором восприимчивости к инфекции, включая «большую тройку» инфекционных заболеваний: ВИЧ/СПИД, туберкулёз и малярия. В данной статье мы обсуждаем современные концепции и разногласия вокруг сложных влияний недоедания на заражение и иммунитет и указываем на возможные последствия борьбы с острым недоеданием. Мы призываем к новым стратегиям по преодолению мировой заболеваемости и смертности при хроническом недоедании в бедных странах и новоявленной угрозы для здравоохранения при переедании в индустриальных странах.
