# Supplementary material for: Malnutrition and Infection: Complex Mechanisms and Global Impacts
Source: PLoS Med. 2007 May 1;4(5):e115. doi: 10.1371/journal.pmed.0040115 (PMC1858706; doi:10.1371/journal.pmed.0040115)
Supplement: Supplementary file 2 [file pmed.0040115.sd002.doc]

La activación y mantenimiento de las respuestas immunitarias durante la infección precisan un incremento del consume energético. La malnutrición energética proteica (MEP) es un factor crítico frecuentemente infravalorado en la susceptibilidad a enfermedades infecciosas, entre otras, las “Tres Grandes” VIH/SIDA, tuberculosis y malaria. En este articulo se discuten conceptos y controversias actuales en torno a la compleja influencia de la malnutrición en la infección y la inmunidad, y señala los prácticos efectos de la medidas contra la malnutrición aguda. Hacemos un llamamiento para atajar globalmente la morbilidad y mortalidad causadas por la malnutrición crónica en países subdesarrollados y la emergente amenaza para la salud publica en países industrializados, la sobrenutrición.
