# Supplementary material for: Malnutrition and Infection: Complex Mechanisms and Global Impacts
Source: PLoS Med. 2007 May 1;4(5):e115. doi: 10.1371/journal.pmed.0040115 (PMC1858706; doi:10.1371/journal.pmed.0040115)
Supplement: Supplementary file 3 [file pmed.0040115.sd003.doc]

L’activation et le maintien de réponses immunitaires aux infections nécessitent une consommation d’énergie accrue. La malnutrition énergétique protéique (MEP) est un facteur primordial, bien que sous-estimé, de sensibilité aux infections, dont les « 3 grandes » maladies, VIH/SIDA, tuberculose et paludisme. Dans cet article nous discutons les concepts et controverses concernant l’influence de la malnutrition sur les infections et l’immunité, en mettant l’accent sur les conséquences des mesures de control de malnutritions graves. Nous en appelons à de nouvelles stratégies pour pallier à la morbidité et à la mortalité mondiale causées par la malnutrition chronique dans les pays pauvres ainsi qu’à la menace émergente pour la santé publique dans les pays industrialises, la surnutrition.
